# Variation in Molar Size and Proportions in the Hominid Lineage: An Inter- and Intraspecific Approach

**DOI:** 10.1093/iob/obae041

**Published:** 2024-11-22

**Authors:** L A D'Addona, V Bernal, P N Gonzalez

**Affiliations:** F acultad de Ciencias Naturales y Museo, Universidad Nacional de La Plata, Buenos Aires CP 1900, Argentina; Consejo Nacional de Investigaciones Científicas y Técnicas (CONICET), Buenos Aires CP C1425FQB, Argentina; Consejo Nacional de Investigaciones Científicas y Técnicas (CONICET), Buenos Aires CP C1425FQB, Argentina; Museo Histórico y Arqueológico “Ricardo Pascual Rosa,” Neuquén Q8320, Argentina; Consejo Nacional de Investigaciones Científicas y Técnicas (CONICET), Buenos Aires CP C1425FQB, Argentina; Estudios en Neurociencias y Sistemas Complejos, Buenos Aires CP 1882, Argentina

## Abstract

The implications of the inhibitory cascade (IC) model in dental diversification have been primarily studied at an interspecific or higher level. In contrast, the study of organisms with recent evolutionary divergence or at an interpopulational scale is still very limited. Here, we assess the effect of changes in molar size and the ratio of local activators to inhibitors on molar proportions based on a compilation of data of crown diameters of the first, second, and third lower and upper molars of extinct and extant hominids and modern human populations. The analysis of allometric changes between the size of each tooth and the size of the molar row shows a negative allometry in first molars (M1), isometric changes in second molars (M2), and a positive allometry in third molars (M3) in both hominin phylogeny and modern human populations. On the other hand, the proportions of lower and upper molars of several hominid species fall outside the morphospace defined by the IC model, while most of the modern human populations fall within the morphospace defined by the model as M1 > M2 > M3. We conclude that there is a phylogenetic structuring for molar size, particularly in the maxilla, with a trend toward mesial-to-distal reduction in the molar row area accompanied by allometric changes. Our findings also show the limitations of the IC model for explaining molar proportions in primates, particularly the variation in the relative size at the interspecific scale in the hominid lineage.

## Introduction

The study of patterns of dental morphological variation—in size and shape—and the evolutionary and developmental processes that originate them at intra- and interspecific levels has significantly contributed to our understanding of the evolutionary history of the hominid lineage (Wolpoff 1971; Kieser 1990; Bailey and Wood 2007; Guatelli-Steimberg 2016). The detailed recording of qualitative and quantitative variables in extant and fossil representatives of hominid species has allowed the description of major changes over the last 7 million years, characterized by a notable reduction in dental size and complexity in the genus *Homo* (Williams and Corruccini 2007; Gómez-Robles et al. 2015; Skinner et al. 2015). In the context of traditional research, dental morphological variation at intra- and interspecific levels has been primarily attributed to the action of microevolutionary processes acting on genetic variation, such as selection, gene flow, and genetic drift (Wolpoff 1971; Guagliardo 1982; Brace et al. 1987; Stojanowski 2004; Hanihara and Ishida 2005; Bernal et al. 2010a, b). On the other hand, the effect of phenotypic plasticity is limited because teeth complete their development within follicles, in a highly protected environment, and after eruption, they are not subject to remodeling, with the influence of environmental factors restricted to changes due to wear and pathological processes. Although the environmental effect cannot be ruled out, it is confined to prenatal or very early developmental stages, while dental formation and mineralization processes take place (Heikkinen et al. 1992; Fearne and Brook 1993; Heikkinen et al. 1994; Jernvall and Jung 2000; Harila-Kaera et al. 2003; Apps et al. 2004).

In recent decades, the molecular and cellular mechanisms that locally regulate the morphogenesis of different dental classes (e.g., incisors, canines, and molars) in the maxilla and mandible have begun to be elucidated (Tummers and Thesleff 2009). These molecular and cellular mechanisms that act during development mediate the action of genetic and environmental factors on the phenotype and, therefore, can limit or favor morphological evolution (i.e., evolvability) in specific trajectories of change (Hendrikse et al. 2007). In this regard, experimental studies in rodents have contributed to understanding the relationship between molecules that locally regulate cell activation and inhibition with resulting dental morphological patterns (Jernvall 2000; Cai et al. 2007; Kavanagh et al. 2007; Ahn et al. 2010; Salazar-Ciudad and Jernvall 2010; Cho et al. 2011). On this basis, Kavanagh and coworkers proposed a model that links developmental mechanisms with the origin and maintenance of morphological variation, and whose expectations can be tested at different levels of evolutionary divergence (Kavanagh et al. 2007). This model, called the inhibitory cascade (IC) model, was based on the *in vitro* culture of the lower first, second, and third molar germs (M1, M2, and M3, respectively) from mouse embryos. The IC model proposes that the proportion of activators and inhibitors regulate the timing of formation and growth of molars, and these proportions are controlled by diet-related natural selection and genetic factors. In particular, the balance between activation and inhibition generates molars of similar size (M3 = M2 = M1), while reducing the inhibitory potential of M1 will favor earlier formation and greater development of the posterior molars, with an increase in inhibitory effect yielding the opposite result.

The implications of the IC model in morphological diversification have been primarily studied at an interspecific or higher taxonomic level with variable results (Polly 2007; Renvoisé et al. 2009; Asahara 2013; Bernal et al. 2013; Halliday and Goswami 2013; Gómez-Robles et al. 2015; Schroer and Wood 2015), indicating that the IC model may not consistently scale with increasing levels of biological organization (Vitek et al. 2020). In contrast, the study of groups of organisms with recent evolutionary divergence or at an interpopulational level is still very limited (as an exception, see Roseman and Delezene 2019). Considering the population level is crucial because the variation arising from modifications in developmental processes (e.g., cell proliferation and differentiation) is fixed or lost through evolutionary processes that operate within and between populations (Jernvall 2000), it is important to take into account this intermediate scale of variation in molar sizes. The aim of this study is to assess the effect of changes in molar size and the ratio of local activators to inhibitors on molar proportions at inter- and intraspecific levels in the hominid lineage, including upper and lower molars. Although the IC model was originally proposed based on molars from a single generation, its application has been extended to other tooth types, such as deciduous teeth and premolars, under the assumption that common processes exist in tooth development. Therefore, it is expected to be applicable to the upper dentition as well (Boughner et al. 2021). In order to achieve this, we analyzed the variation in size and proportions of the upper and lower molars from a set of fossil and extant hominids and modern human populations, based on published dental measurements. Crown diameters were used for obtaining the area of each molar, as an estimation of molar size, while the ratio between molar areas was used to estimate the proportions to test the predictions derived from the IC model of dental development (Kavanagh et al. 2007). Previous studies analyzing molar size in primates and modern humans have not fully aligned with the predictions of the IC model (Bernal et al. 2013; Roseman and Delezene 2019; Boughner et al. 2021). It remains uncertain whether this inconsistency reflects a limitation of the model itself or a particular characteristic of primates as a study group (for an in-depth discussion, see Chapple and Skinner 2023). In this study, we aim to contribute to this issue by using an extensive sample that captures variations in molar size over extended evolutionary timescales, encompassing both modern primates and fossil hominins.

## Materials and methods

### Samples

We compiled a database of buccolingual (BL) and mesiodistal (MD) crown diameters of the first, second, and third lower and upper molars of fossil hominins, extant specimens of *Gorilla* and *Pan* (Table 1, and additional data, including the complete bibliography, are included in Supplementary Tables 1 and 3; *N* = ∼1100 teeth) and 56 populations of modern humans from worldwide distributions (additional data, including the complete bibliography, are included in Supplementary Table 2; *N* = 27,064 teeth). Some of the species (i.e., *Australopithecus deyiremeda* and *Homo floresiensis*) and populations (one from North America, one from Oceania, two from Europe, and three from Asia) studied for this compilation did not have molar data for the maxilla. In this study, we refer to “hominids” in the analyses that include both fossil hominins of the phylogenetic lineage of *Homo sapiens* and extant specimens of the species of *Gorilla, Pan*, and *Homo sapiens*. We refer to “hominins” in the analyses that only include the evolutionary lineage of *H. sapiens* and populations of modern humans.

**Table 1.** Species analyzed in the hominid lineage

**ID**	**Species**	***N*** ^a^
1	*Australopithecus anamensis*	30
2	*Australopithecus afarensis*	33
3	*Australopithecus africanus*	32
4	*Australopithecus deyiremeda*	1
5	*Australopithecus sediba*	2
6	*Homo habilis*	23
7	*Homo erectus (*Asia)	57
8	*Homo ergaster* (Africa)	15
9	*Homo georgicus* (Dmanisi)	6
10	*Homo naledi*	2
11	*Homo floresiensis*	2
12	*Homo heidelbergensis*	38
13	*Homo neanderthalensis*	90
14	*Homo sapiens*	5663^b^
15	*Paranthropus boisei*	5
16	*Paranthropus robustus*	63
17	*Gorilla beringei*	12
18	*Gorilla gorilla*	47
19	*Pan paniscus*	25
20	*Pan t. troglodytes*	56
21	*Pan t. schweinfurthii*	26

^a^ *N*: number of specimens analyzed.

^b^Total of individuals from the 56 human populations detailed in Supplementary Table 2. Additional data, including the complete bibliography, are included in Supplementary Tables 1 and 3.

The molar size (i.e., area) was calculated as the product of BL and MD diameters, which were averaged per species or population. The proportions M3/M1 and M2/M1 were estimated from the respective molar size. The variables obtained correspond to one side or the average of both sides, depending on the availability in the literature. To obtain the molar row size, the areas of the three molars were summed (M1 + M2 + M3).

### Molar size and proportions

Comparisons of the size of lower and upper M1, M2, and M3 among different species were conducted to identify interspecific dental patterns. Evolutionary changes in the molar row size of the mandible and maxilla of hominid species were visualized using the method developed by Revell (2013), mapping characters onto a phylogenetic tree previously obtained by Rocatti and Perez (2019). This tree was obtained using molecular and craniodental data from fossil and extant hominids (Perelman et al. 2011; Dembo et al. 2015; Rocatti and Perez 2019). The analysis was performed using the contMap function from the phytools package developed by Revell (2012) for R (R Core Team 2014). To assess the phylogenetic structure of molar size in the hominid phylogeny, a phylogenetic signal analysis was conducted. K values (used as an estimator of the phylogenetic signal strength) were estimated for the molar row size, molar size, and M2/M1 and M3/M1 proportions following the approach of Blomberg et al. (2003). These analyses were performed using the picante package (Kembel et al. 2010) for R (R Core Team 2014). Finally, the phylogenetic generalized least-squares regression method (PGLS; Rohlf 2001) was employed to assess the association of molar size and molar proportions (M2/M1 and M3/M1) with the complete molar row size. This method considers the lack of independence in the data due to shared evolutionary history by introducing a covariance matrix derived from the clade's phylogeny into the error term of the regression model. The size of the molar area was transformed to logarithm. Regression models were fitted using the CAPER package developed by Orme et al. (2013) for R (R Core Team 2014). The confidence intervals of 95% of the slopes obtained from the regression models were also estimated in R.

At an intraspecific level, changes in the dental pattern of the three upper and lower molars were evaluated by comparing molar sizes between populations. To assess the association of molar size with row size in human populations, a spatial simultaneous autoregressive lag model estimation (lagSAR) by maximum likelihood was conducted. This analysis accounts for the lack of independence in the data due to their spatial distribution derived from the evolutionary history of populations. This assumes greater similarity between nearby populations and an increase in differences as geographic distance increases, influenced by mechanisms such as drift, migration, gene flow, and environmental factors. In this context, the regression model incorporates an error term in the covariance matrix that models the spatial correlation structure (Diniz-Filho et al. 2009; Perez et al. 2010). The elements of this matrix were estimated by the inverse of the geodesic distance between populations and were fitted using the package “spatialreg” version 1.3-3 for R.

### Evaluation of the inhibitory cascade model

The intra- and interspecific variation in molar proportions was evaluated according to the IC model (Kavanagh et al. 2007). The mathematical formulation of this model suggests that the relative size of the lower molars results from an inhibitory cascade through the molars and can be described as *Y* = 1 + [(*a* − *i*)/*i*] (*X* − 1), where *Y* is the molar area relative to its position, *X* represents the molar position (i.e., 1, 2, or 3), *a* is the activator, and *i* is the inhibitor. The term (*a* − *i*/*i*) represents the relative magnitude of activators versus inhibitors. Molar areas are derived from the equation according to the following formula: M1 = 1, M2 = *a*/*i*, and M3 = 2*a*/*i* − 1. From these formulas, the relationship between the proportions M2/M1 and M3/M1 can be expressed as M3/M1 = 2 (M2/M1) − 1. This allows the comparison of observed relative size variation with values predicted by the model. Two main expectations can be derived from the model: (1) if a linear model is fitted to the proportions M2/M1 and M3/M1, a line with a slope of 2 and an intercept of −1 is obtained; and (2) M2 corresponds to one-third of the size of the complete molar row (Kavanagh et al. 2007).

## Results

### Variation in molar size

**Fig. 1:**
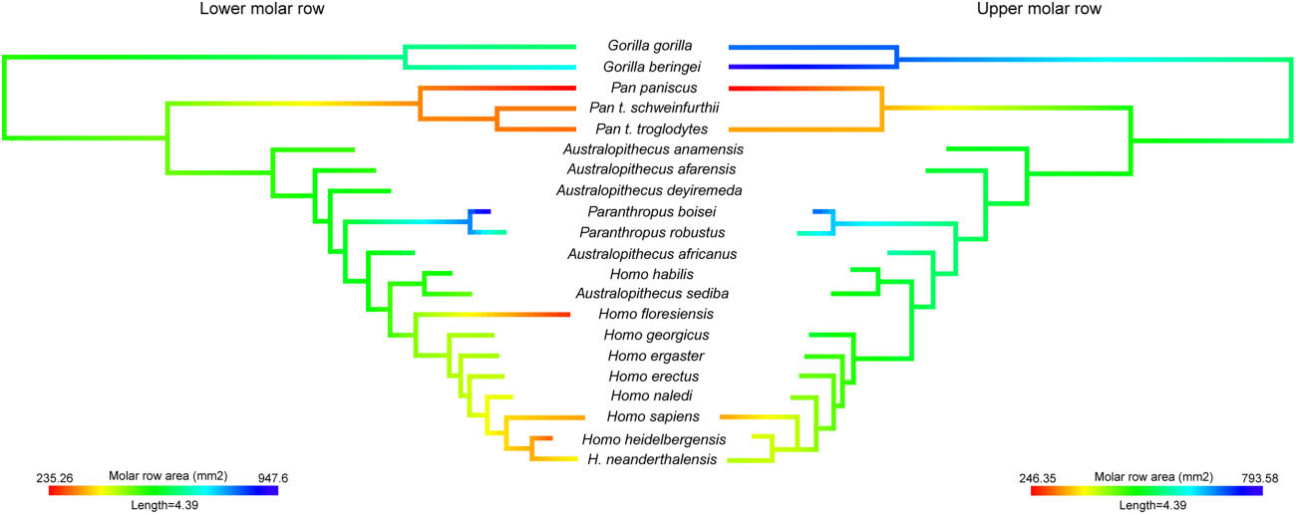
Size of the lower and upper row molar mapped onto the phylogeny of hominids.

In the interspecific analysis, a trend toward dental reduction within the phylogeny of hominin species was observed. Concerning the size of the lower molar row, *Pan, Homo floresiensis, H. neanderthalensis, H. heidelbergensis*, and *H. sapiens* exhibited the smallest size. The remaining *Homo* species, as well as *Australopithecus* and *Gorilla*, showed similar size. Lastly, *Paranthropus* displayed the largest size (Fig. 1). The size of the upper molar row displayed a similar trend, although in this case the *Gorilla* exhibited the largest size along with *Paranthropus* (Fig. 1).

**Fig. 2:**
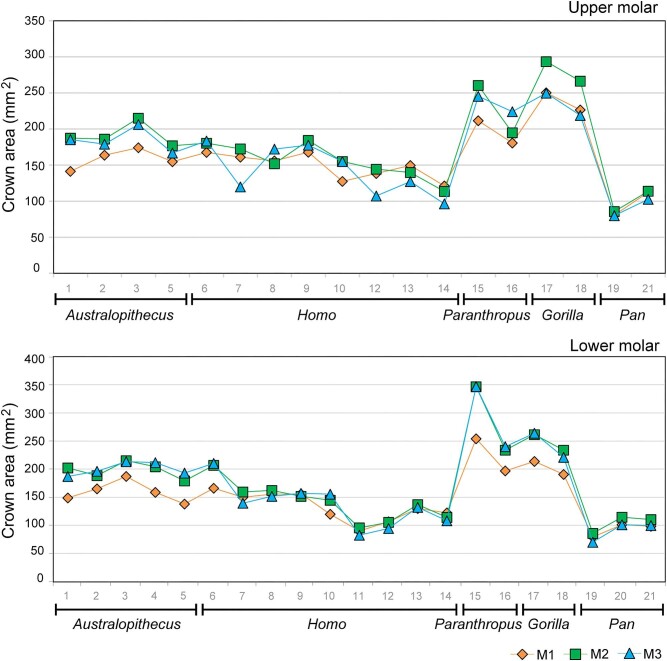
Mean molar crown area (in mm²) of hominid species for the maxilla and mandible. M1 (diamonds), M2 (squares), M3 (triangles). The numbers in the x-axis correspond to species within each genus (see references in Table 1). M: molar.

The size of the molars follows a similar pattern in the species of *Australopithecus, Paranthropus*, and *Gorilla*, with the M1 exhibiting a smaller size than the other two molars, except for the maxilla of *Gorilla beringei* (Fig. 2). Likewise, a general trend observed for both *Pan* and *Homo* is that they maintain the relative size of the M1 compared to what is observed in *Australopithecus*, while the posterior molars (M2 and M3) tend to show a reduction in size relative to their M1.

Regarding the association between the variation in the individual molar size and the molar row size, a similar trend can be observed for both upper and lower molars. The results of OLS and PGLS analyses indicate a negative allometry for M1 and isometry for M2 associated with changes in the size of the row (Table 2). The third molar shows a positive allometry in the mandible and isometry in the maxilla (when considering the 95% confidence intervals).

**Table 2.** Interspecific variation in molar size relative to molar row size through linear regression (OLS) and phylogenetic regression (PGLS)

	**Molar**	***R* ^2^**	**Slope** ^a^
Mandible	M1	0.956*	OLS: 0.818 (0.739/0.897) PGLS: 0.814 (0.730/0.897)
	M2	0.989*	OLS: 1.010 (0.967/1.053) PGLS: 1.014 (0.964/1.064)
	M3	0.987*	OLS: 1.157 (1.097/1.216) PGLS: 1.158 (1.096/1.220)
Maxilla	M1	0.920*	OLS: 0.854 (0.733/0.976) PGLS: 0.847 (0.715/0.979)
	M2	0.975*	OLS: 1.041 (0.957/1.125) PGLS: 1.040 (0.952/1.127)
	M3	0.924*	OLS: 1.101 (0.940/1.263) PGLS: 1.110 (0.942/1.279)

^a^Confidence intervals (95%) are indicated in parentheses.

*P < 0.001.

**Fig. 3:**
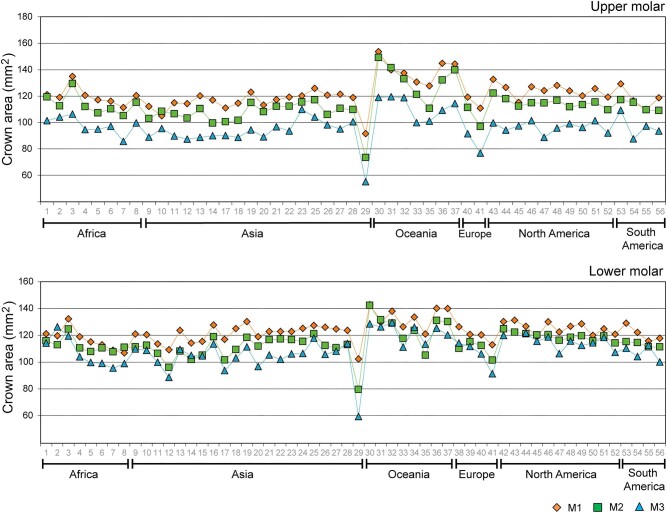
Mean molar crown area (in mm²) for the maxilla and mandible of human populations. M1 (diamonds), M2 (squares), and M3 (triangles). The numbers in the x-axis correspond to modern populations summarized in Supplementary Table 1. M: molar.

The analysis of molar size at an interpopulational level in *H. sapiens* shows that groups originating from Oceania have the largest size, and generally, populations from Asia have the smallest size, although there is a significant variation within each continent (Fig. 3). Regarding lower molars, 43 out of the 56 analyzed populations (76.8%) exhibited a reduction in size with anteroposterior direction from M1 to M3, showing a pattern of M1 > M2 > M3. Of the remaining, 8 populations (14.3%) showed an increase in M3 size, surpassing the size of M2 but not that of M1, presenting a pattern of M1 > M2 < M3. Conversely, 3 populations (5.3%) showed an increase in M2 size, with M2 being the largest molar, presenting a pattern of M1 < M2 > M3. Finally, a single population from Africa showed a significant increase in M3 size, with M3 being the largest molar, presenting a pattern of M1 < M2 < M3. Finally, one population (1.8%) from North America had no information of size for M3 (Fig. 3). Regarding upper molars, 47 out of 49 analyzed populations (95.9%) displayed a trend of reduction in size with anteroposterior direction from M1 to M3, with a pattern of M1 > M2 > M3. The remaining 2 populations (4.1%) showed an increase in M2 size, with a pattern of M1 < M2 > M3 (Fig. 3).

Regarding the association between row size and the variation in the individual molar size, a similar trend was observed in both mandible and maxilla. M1 exhibited a negative allometry, M2 showed isometric changes, and M3 displayed a positive allometry (Table 3).

**Table 3.** Human population variation in molar size relative to molar row size through linear regression (OLS) and spatial regression (lagSAR)

	**Molar**	***R* ^2^**	**Slope** ^a^
Mandible	M1	0.835*	OLS: 0.724 (0.637/0.812) lagSAR: 0.665 (0.582/0.747)
	M2	0.920*	OLS: 0.990 (0.910/1.069) lagSAR: 0.987 (0.902/1.072)
	M3	0.909*	OLS: 1.366 (1.249/1.484) lagSAR: 1.397 (1.276/1.518)
Maxilla	M1	0.900*	OLS: 0.830 (0.750/0.910) lagSAR: 0.771 (0.693/0.848)
	M2	0.960*	OLS: 1.065 (1.002/1.128) lagSAR: 1.067 (0.998/1.135)
	M3	0.896*	OLS: 1.156 (1.042/1.270) lagSAR: 1.199 (1.087/1.311)

^a^Confidence intervals (95%) are indicated in parentheses.

*P < 0.001.

### Variation in molar proportions and fit to the inhibitory cascade model

**Fig. 4:**
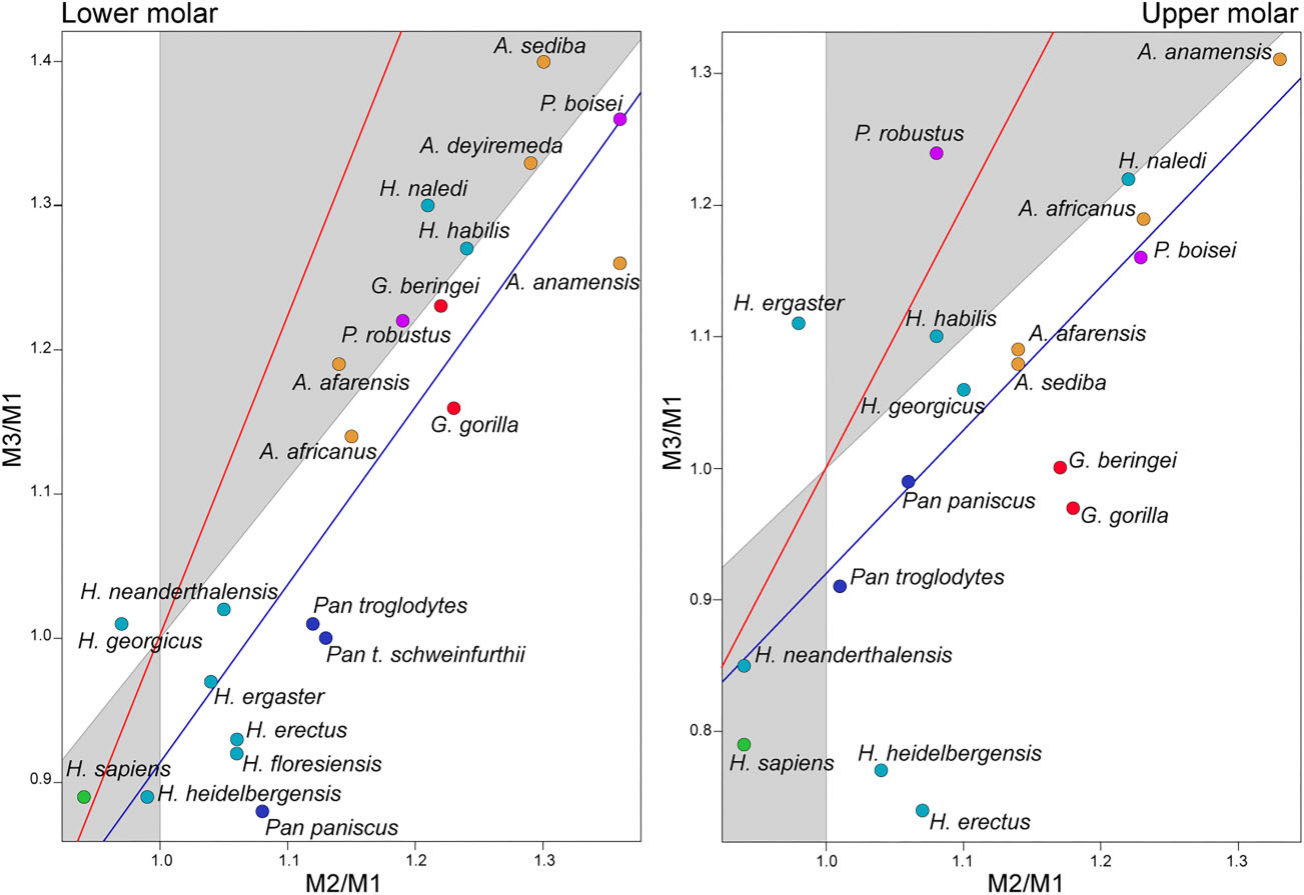
Fit of molar proportions to the IC model in the hominid species. Red line: IC model line (slope: 2, intercept: −1); blue line: fit of phylogenetic regression (lower molars: slope: 1.23, intercept: −0.32; upper molars: slope: 1.09, intercept: −0.17).

At the interspecific level, the pattern of molar proportions of the lower and upper molars does not show a good fit with the predictions of the IC model (Fig. 4). For the lower molars, a slope value of 1.23 (with a 95% confidence interval between 0.93 and 1.53) and an intercept of −0.32 (with a 95% confidence interval between −0.68 and 0.04) were obtained, while for the upper molars, the slope value was 1.09 (with a 95% confidence interval between 0.51 and 1.67) and the intercept was −0.17 (with a 95% confidence interval between −0.97 and 0.33). Despite the suboptimal fit, lower molars of *H. sapiens, H. naledi, H. heidelbergensis, H. habilis, A. sediba, A. deyiremeda, A. afarensis*, and *P. robustus* fall within the morphospace defined by the model as a result of the action of activating and inhibiting molecules (M1 < M2 < M3 or M1 > M2 > M3). However, the lower molars of the remaining hominid species fall outside the space defined by the model. For upper molars, it can be observed that the species *H. sapiens, H. naledi, H. habilis, H. neanderthalensis*, and *P. robustus* fall within the morphospace region defined by the increase or decrease of the inhibitory effect (M1 < M2 < M3 or M1 > M2 > M3). The upper molars of the remaining hominid species fall outside the region defined by the IC model.

With respect to the second prediction of the model, we observed that the mean value of the M2 size proportion was close to 33.33%, with a maximum value of 38.07% (represented by the upper M2 of *H. erectus*) and a minimum value of 31.68% (represented by the upper M2 of *H. ergaster*) (Supplementary Table 4).

**Fig. 5:**
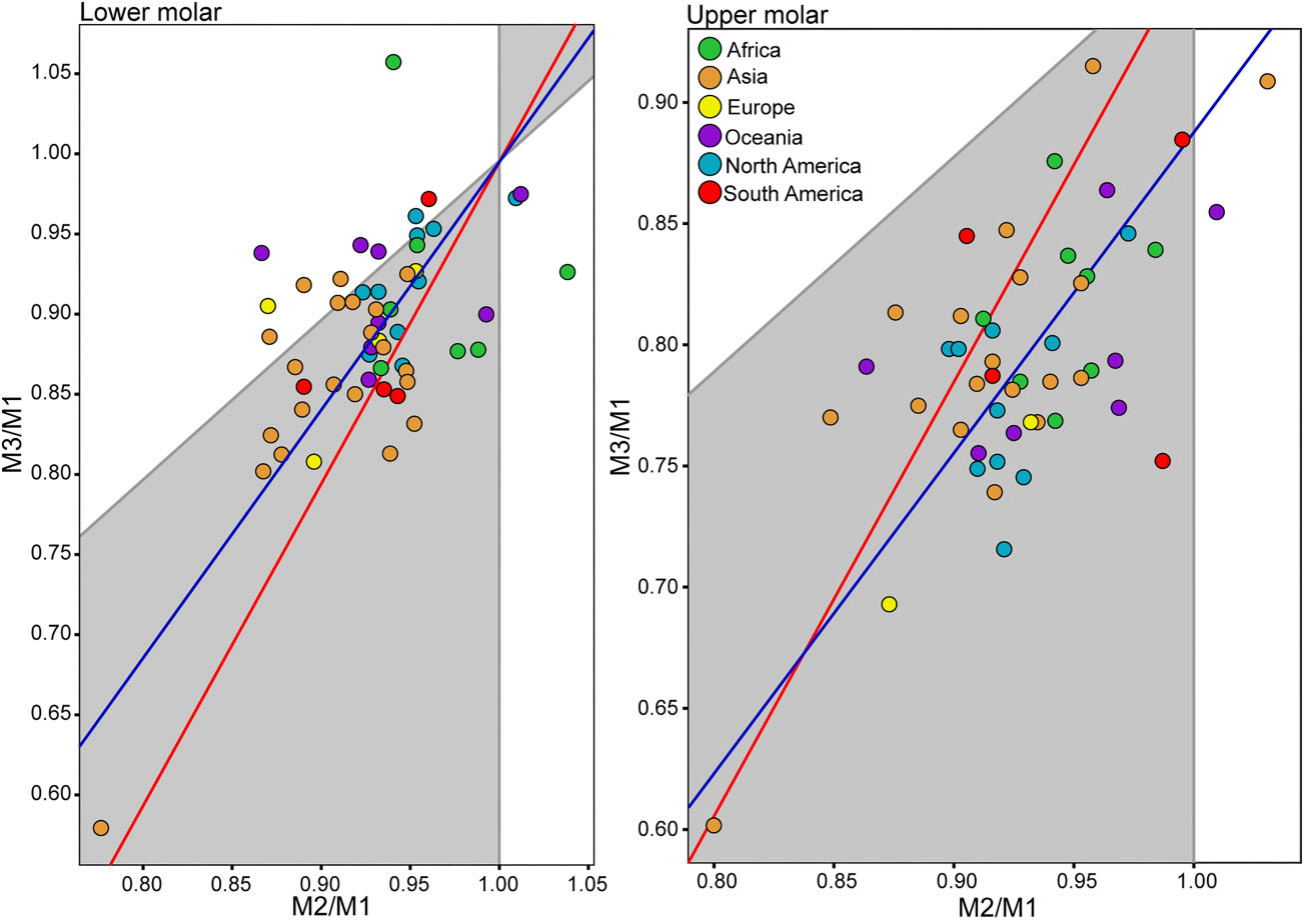
Fit of proportions to the IC model in human populations. Dot colors indicate the continent of origin. Red line: IC model line (slope: 2, intercept: −1); blue line: fit of spatial regression (lower molars: slope: 1.53, intercept: −0.55; upper molars: slope: 1.47, intercept: −0.58).

The analysis of molar proportions (M2/M1 and M3/M1) for human populations indicates that it is not possible to statistically reject the hypothesis of fit to the IC model given that the 95% confidence intervals were very wide (Fig. 5). For lower molars, a slope of 1.53 (with a 95% confidence interval between 0.81 and 2.28) and an intercept of −0.55 (with a 95% confidence interval between −1.24 and 0.11) were obtained, while for upper molars, the slope was 1.47 (with a 95% confidence interval between 0.69 and 2.51) and the intercept was −0.58 (with a 95% confidence interval between −1.56 and 0.12). As observed in Fig. 5, the values corresponding to human populations reflect a distribution close to the region of equilibrium between activating and inhibiting molecules, with similar sizes among the three molars (M1 = M2 = M3).

With regard to the prediction about the proportion of M2, modern human populations conform to the expected value of 33%, with values ranging from a maximum of 34.51% (represented by the upper M2 of Oceania) to a minimum of 32.89% (represented by the lower M2 of Asia) (Supplementary Table 5).

## Discussion

The size of upper and lower molars in modern human populations results from a reduction that occurred in the hominin lineage in an MD direction, where the most mesial tooth is characterized by a larger size and less variation, while the most distal tooth exhibits a smaller size and greater variation (Butler 1939; Kieser 1990; Bermúdez de Castro and Nicolas 1995; Gómez-Robles and Polly 2012; Evans et al. 2016). Recently, the variation in molar size has been explained as the result of the action of activating/inhibiting molecules acting locally on molar development, where the size of mesial teeth has an inhibitory effect on posterior dentition, while the tissue surrounding the developing dental germs has an activating effect on development (Jernvall 2000; Cai et al. 2007; Kavanagh et al. 2007; Ahn et al. 2010; Salazar-Ciudad and Jernvall 2010; Cho et al. 2011). Based on an extensive literature review, this study evaluated changes in molar size and their relationship with changes in molar row size for upper and lower dentition contrasting between hominid species and modern human populations. The results obtained partially support the inhibitory cascade hypothesis in the case of hominids, with a better fit observed in human populations.

### Allometric changes

The analysis of allometric changes between the size of each tooth and the size of the molar row shows an allometric component influencing the variation in the size of upper and lower molars at both inter- and intraspecific levels. A general trend was observed where M1 exhibited a negative allometry both in hominin phylogeny and in modern human populations, indicating a decrease in the relative size of M1 with increasing molar row size. This relationship suggests that part of the variation in the relative size of M1 among species and populations is generated by changes associated with variation in molar row size. Conversely, changes observed in M2 were isometric with respect to molar row size. Lastly, a positive allometry was observed in M3 in both scales of analysis, except for the upper molar at the intraspecific level, indicating that an increase in molar row size is associated with an increase in the relative size of M3. This general trend is primarily observed for the mandible and, to a lesser extent, for the maxilla. It is noteworthy that the allometric coefficient was higher for M3, indicating a greater effect of changes in posterior dentition size on this molar. Furthermore, the general trend observed in molar size throughout hominin evolution was characterized by a transition from a primitive pattern of M1 < M2 < M3 to a derived pattern of M1 > M2 > M3, where the size of M1 showed relatively less variation, while the size of the two posterior molars exhibited greater differences among hominids. Specifically, the genera *Paranthropus* and *Australopithecus* exhibited a similar relative size pattern, characterized by M1 < M2 ∼ M3, even though they differed in the absolute size of the molar row. The pattern of relative size changed in the *Homo* genus, with older species showing a reduction mainly in M3 (M1 < M2 > M3), culminating in *H. sapiens* with a reduction in M2 along with an even greater decrease in M3 (M1 > M2 > M3). In the interpopulation analysis, this MD reduction was recorded for most modern human populations studied, being more pronounced in the maxilla.

The overall trends described in this study have been previously observed at both interspecific (Wolpoff 1971; Bermúdez de Castro and Nicolas 1995; Bailey and Wood 2007; Gómez-Robles et al. 2011; Gómez-Robles and Polly 2012; Gómez-Robles et al. 2015; Billet and Bardin 2021) and intra-specific levels (Wolpoff 1971; Bailey and Wood 2007; Evans et al. 2016; Morita et al. 2016; Bermúdez de Castro et al. 2021). The relatively less variation of M1 compared to the posterior teeth has been previously observed in allometric assessments with body size (Gingerich et al. 1982; Billet and Bardin 2021), where the greater stability of M1 is demonstrated by its lesser variation when adjusted for body mass, while M3 shows greater variation, exhibiting a smaller size in organisms with a lower body mass rate. In the same vein, Gómez-Robles and Polly (2012) observed for a large sample of hominids an integration of the molars into a module independent of the rest of the dentition, where the coevolution of the three molars tends toward the reduction of the distal area. Similarly, in human populations, the formation time of each tooth has been proposed as an underlying cause of the observed allometric pattern in molars, the greater variability of M3 (Evans et al. 2016; Bermúdez de Castro et al. 2021), and the relatively lower variation of M1 (Morita et al. 2016; Renaud et al. 2017). Indeed, while M1 begins its development during the embryonic period, M2 and M3 start their development after birth, with considerably longer formation times, making them more exposed to epigenetic or environmental processes that could interfere with development (Morita et al. 2016; Renaud et al. 2017; Bermúdez de Castro et al. 2021). On the other hand, it has been suggested that the reduction in the relative size of molars would be associated with the overall reduction in crown size due to lower crown complexity related to the loss of cusps (Wolpoff 1971; Bailey and Wood 2007).

### Inhibitory cascade model

The results obtained partially support the hypothesis that relative molar size in the mandible and maxilla is produced by changes in the ratio of activators/inhibitors in the case of hominids, with a better fit observed in human populations.

The proportions of lower and upper molars of several species of the genera *Homo, Australopithecus*, and *Paranthropus* fall within the morphospace defined by the IC model, with a pattern characterized by M1 < M2 < M3 or M1 > M2 > M3. However, approximately half of the analyzed species were located outside of this morphospace, with dental patterns of M1 < M2 > M3. An exception were the proportions of lower molars of *H. georgicus*, which occupied the region of morphospace defined by the pattern M1 > M2 < M3 as well as upper molars of *H. ergaster*. The lack of consistency between molar proportions in hominids and the values predicted by the IC model by changes in the ratio of activators and inhibitors aligns with previous studies, both for hominoids and cercopithecoids (Schroer and Wood 2015; Carter and Worthington 2016; Roseman and Delezene 2019).

These departures from the IC model observed in hominids may originate from the model itself, which, by focusing solely on relative size changes associated with activation and inhibition factors, limits the consideration of other factors, such as the influence of static allometry (Machado et al. 2023). Specifically, we observed an allometric component influencing the variation in the size of upper and lower molars at both inter- and intraspecific levels, with a consistent trend in hominids and modern human populations, despite differences in the fit to the IC model between both levels of analysis. Other influential factors to consider are the differences in dental maturation times between species. The trend toward a reduction in the size of M2 and M3 could be linked to differences in the development and exposure timing of each tooth, relative to M1. A longer exposure time of each tooth compared to the other molars may result in greater inhibition in development (Schroer and Wood 2015), while delayed development can postpone dental growth, leading to a reduction in overall molar size, as well as in their cusps (Jernvall 2000; Gómez-Robles et al. 2015). Even the sources of exposure may expand to include other teeth, such as premolars (Evans et al. 2016), although the selective pressures experienced by molars and premolars differ due to development and eruption timing, as well as the alveolar space each occupies (i.e., where premolars replace deciduous molars while permanent molars develop behind them; Hlusko et al. 2016).

Similarly, quantitative genetic studies reinforce the importance of recognizing the genetic mechanisms underlying dental morphological variation, supported by an incomplete pleiotropy between the premolar module and the molar module (Hlusko et al. 2006, 2011; Gómez-Robles and Polly 2012; Schroer and Wood 2015; Hlusko et al. 2016; Brasil et al. 2020). Navarro and Maga (2018) argue that there is hereditary variation in the inhibitory cascade, where the main developmental genes would explain both the variation in tooth size and the control of the activation/inhibition balance among successively developing molars. In this sense, a greater or lesser correlation between molars and the total row size will depend on whether they are under the same directional selection, which would in turn limit inherited variation through canalization (Navarro and Maga 2018). As some alternatives to the IC model have been proposed, it is necessary to consider sources of molar size covariation that do not arise from signaling mechanisms, but from differences in the integration and canalization of genetic and environmental factors throughout development (Roseman and Delezene 2019). Likewise, functional aspects associated with proper occlusion between the maxilla and mandible, species-specific growth rates, and available maxillary space (Boughner and Dean 2004; Labonne et al. 2014; Hlusko et al. 2016; Sadier et al. 2023) should be considered. In this sense, different processes could lead to a similar pattern of variation in molar morphology through alternative pathways of covariation and evolution than those contemplated in the IC model.

Unlike what was observed in the interspecific analysis, the results of the analysis of molar proportion variation in modern human populations showed a good fit to the IC model for both maxilla and mandible, supporting the explanation of molar proportion variation by alteration of the balance between activators and inhibitors. When considering the confidence intervals of the regression between molar proportions, the results were consistent with the intercept and slope values predicted by the IC model, although this result should be interpreted with caution due to the wide confidence intervals found at the intraspecific level here as well as in previous studies (Bernal et al. 2013). Nevertheless, the M2/M1 and M3/M1 proportions of both maxilla and mandible in most populations fall within the morphospace defined by the model as M1 > M2 > M3. Furthermore, the analysis of molar proportions at the population level showed that the distribution of populations tends to be less dispersed for the maxilla compared to the mandible. Recent observations indicate a higher sensitivity to local factors in the maxilla as opposed to the mandible (Boughner et al. 2021). Although classical observations propose that teeth have relatively independent development than bone tissues, both structures exhibit spatial integration (Atchley and Hall 1991), where the tissue surrounding the molar generates and releases different signals and molecules activating the development of dental structures (Matalova et al. 2015). In fact, for modern humans, it has been observed that teeth developing later in ontogeny are more constrained in space and tend to be smaller (Takahashi et al. 2007). Takahashi et al. (2007) even observed that the trend repeats among cusps of the same molar; those that develop first are the relatively less variable (protocone) and show an increase in size at the expense of cusps that develop later (mainly the hypocone).

Regarding the IC model prediction that M2 represents 33% of the molar row size, the results obtained at the interpopulation level met the expectation, while at the interspecies level, the results showed higher values. These findings align with the isometric changes observed in M2 concerning the entire molar row, meaning that the proportion of the M2 area remains constant. Based on the fitting of molar areas to this expectation of the IC model, Evans et al. (2016) emphasize the importance of the activation/inhibition mechanism in regulating the relative size pattern of postcanine dentition in hominins. According to these authors, even in species located outside the region of the IC model, molar proportions are influenced by the system of activating and inhibiting molecules, although outside the balance zones (Evans et al. 2016). In other words, a relatively large M1 can generate greater inhibition on M2, which would reduce its relative size, and consequently, generate less inhibition on M3, which would increase its relative size, thus obtaining the pattern M1 > M2 < M3 (as observed in the upper molars of *H. ergaster*). Additionally, Bermúdez de Castro et al. (2021), although observing limited application of the IC model to lower molar size variation, also find that the prediction that M2 is 33% of the molar row size holds. However, based on their observations regarding the development time of M3, the authors caution that the IC model and the size gradient seem to influence more directly both M1 and M2 than M3 (Bermúdez de Castro et al. 2021).

In summary, the results obtained in this study emphasize the complexity of the processes regulating molar development in the emergence of morphological variation at inter- and intraspecific levels within the hominid lineage. Overall, molar size demonstrates a consistent trend toward mesial-to-distal reduction in the molar row area accompanied by allometric changes, especially in the maxilla. Conversely, the lack of correspondence between hominids and modern human populations for relative size changes associated with inhibition/activation factors supports the limitations observed in the IC model in primates. The allometric variation observed in this study reinforces the premise that factors affecting the entire organism also impact dental development and must be considered alongside local regulatory factors. In this regard, a joint analysis of intrinsic mechanisms that regulate morphological differentiation and growth, and systemic factors that control growth, is essential to understand the extent to which the integration of both signaling pathways produces the observed dental morphological variation.

## Supplementary Material

obae041_Supplemental_Files
