# Supplementary material for: Variation in Molar Size and Proportions in the Hominid Lineage: An Inter- and Intraspecific Approach
Source: Integr Org Biol. 2024 Nov 22;6(1):obae041. doi: 10.1093/iob/obae041 (PMC11631436; doi:10.1093/iob/obae041)
Supplement: obae041_Supplemental_Files [file obae041_supplemental_files.zip › Supplementary Table 4.docx]

Supplementary Table 4. Percentage of the area of M2 for the hominid species. Confidence intervals are shown between parentheses (for samples with more than three specimens).

| **Hominins** | **Lower M2 %** | **Upper M2 %** |
| --- | --- | --- |
| *Australopithecus anamensis* | 37.56 (36.93 / 39.07) | 36.46 (35.29 / 36.71) |
| *Australopithecus afarensis* | 34.31 (33.28 / 34.72) | 35.18 (34.54 / 35.46) |
| *Australopithecus africanus* | 34.96 (34.47 / 35.53) | 36.10 (34.91 / 37.09) |
| *Australopithecus deyiremeda* | 35.54 | - |
| *Australopithecus sediba* | 35.09 | 35.48 |
| *Homo habilis* | 35.43 (34.03 / 35.97) | 33.97 (32.77 / 35.23) |
| *Homo erectus* | 35.51 (35.60 / 36.40) | 38.07 (36.05 / 39.95) |
| *Homo ergaster* | 34.54 (34.20 / 35.80) | 31.68 (30.09 / 33.91) |
| *Homo georgicus* | 32.64 (30.26 / 35.74) | 34.79 (29.29 / 40.71) |
| *Homo naledi* | 34.45 | 35.42 |
| *Homo floresiensis* | 35.66 | - |
| *Homo heidelbergensis* | 34.43 (33.39 / 34.61) | 37.01 (35.73 / 38.27) |
| *Homo neanderthalensis* | 34.33 (33.64 / 34.36) | 33.58 (33.54 / 34.46) |
| *Homo sapiens* | 33.14 (32.81 / 33.19) | 34.23 (33.82 / 34.18) |
| *Paranthropus boisei* | 36.58 (34.54 / 39.46) | 36.31 |
| *Paranthropus robustus* | 34.86 (34.55 / 35.45) | 32.51 (32.40 / 33.60) |
| *Gorilla beringei* | 35.36 (34.16 / 35.84) | 36.99 |
| *Gorilla g gorilla* | 36.22 (35.54 / 36.46) | 37.42 |
| *Pan paniscus* | 36.51 (36.15 / 37.85) | 34.78 |
| *Pan t schweinfurthii* | 36.13 (35.48 / 36.52) | 35.18 |
| *Pan t troglodytes* | 35.78 (35.65 / 36.35) | 34.62 |
