# Supplementary material for: Variation in Molar Size and Proportions in the Hominid Lineage: An Inter- and Intraspecific Approach
Source: Integr Org Biol. 2024 Nov 22;6(1):obae041. doi: 10.1093/iob/obae041 (PMC11631436; doi:10.1093/iob/obae041)
Supplement: obae041_Supplemental_Files [file obae041_supplemental_files.zip › Supplementary Table 5.docx]

Supplementary Table 5. Percentage of the area of M2 for the human populations. Confidence intervals, estimated by region, are shown between parentheses (for samples with more than three specimens).

| **Region** | **Lower M2 %** | **Upper M2 %** |
| --- | --- | --- |
| Africa | 33.45 (31.93 / 34.07) | 34.31 (32.97 / 35.03) |
| Asia | 32.89 (32.68 / 33.32) | 33.87 (33.63 / 34.37) |
| Oceania | 33.00 (32.11 / 33.89) | 34.51 (33.11 / 34.89) |
| Europe | 32.72 (31.60 / 34.40) | 34.34 |
| North America | 33.16 (32.43 / 33.57) | 34.22 (33.02 / 34.98) |
| South America | 33.17 (31.99 / 34.01) | 34.36 (31.92 / 36.08) |
